# The feasibility and acceptability of mass drug administration for malaria in Cambodia: a mixed-methods study

**DOI:** 10.1093/trstmh/try053

**Published:** 2018-06-16

**Authors:** Thomas J Peto, Rupam Tripura, Nou Sanann, Bipin Adhikari, James Callery, Mark Droogleever, Chhouen Heng, Phaik Yeong Cheah, Chan Davoeung, Chea Nguon, Lorenz von Seidlein, Arjen M Dondorp, Christopher Pell

**Affiliations:** 1 Mahidol-Oxford Tropical Medicine Research Unit, Faculty of Tropical Medicine, Mahidol University, Bangkok, Thailand; 2 Centre for Tropical Medicine and Global Health, Nuffield Department of Clinical Medicine, University of Oxford, Oxford, UK; 3 Academic Medical Centre, University of Amsterdam, Amsterdam, The Netherlands; 4 Battambang Provincial Health Department, Mohatep Street, Battambang, Cambodia; 5 National Centre for Parasitology, Entomology and Malaria Control, 477 Betong, Khan Sen Sok, Phnom Penh, Cambodia; 6 Centre for Social Science and Global Health, University of Amsterdam, Amsterdam, The Netherlands; 7 Amsterdam Institute for Global Health and Development, Amsterdam, The Netherlands

**Keywords:** malaria elimination, mass drug administration, Southeast Asia

## Abstract

**Background:**

Mass drug administrations (MDAs) are part of the World Health Organization’s *Plasmodium falciparum* elimination strategy for the Greater Mekong Subregion (GMS). In Cambodia, a 2015–2017 clinical trial evaluated the effectiveness of MDA. This article explores factors that influence the feasibility and acceptability of MDA, including seasonal timing, financial incentives and the delivery model.

**Methods:**

Quantitative data were collected through structured questionnaires from the heads of 163 households. Qualitative data were collected through 25 semi-structured interviews and 5 focus group discussions with villagers and local health staff. Calendars of village activities were created and meteorological and malaria treatment records were collected.

**Results:**

MDA delivered house-to-house or at a central point, with or without compensation, were equally acceptable and did not affect coverage. People who knew about the rationale for the MDA, asymptomatic infections and transmission were more likely to participate. In western Cambodia, MDA delivered house-to-house by volunteers at the end of the dry season may be most practicable but requires the subsequent treatment of in-migrants to prevent reintroduction of infections.

**Conclusions:**

For MDA targeted at individual villages or village clusters it is important to understand local preferences for community mobilisation, delivery and timing, as several models of MDA are feasible.

## Background

Failure to stop the spread of multidrug-resistant *Plasmodium falciparum* parasites^1–4^ across and out of the Greater Mekong Subregion (GMS) could damage international malaria control and jeopardise recent declines in malaria-related deaths.^5^ In addition to appropriate case management and the distribution of long-lasting insecticide-treated bed nets (LLINs), more intensive interventions to interrupt transmission are under evaluation. One of these is mass drug administration (MDA), the antimalarial treatment of an entire population, regardless of infection status, in a defined geographic location.^6–11^

A targeted approach to malaria elimination (TME), which included MDA, appropriate case management and the distribution of LLINs, was evaluated between 2013 and 2017 across the GMS.^12–24^ MDA consisted of three rounds, given over 3 months, of the antimalarial dihydroartemisinin–piperaquine (DHA-PPQ).^5^ The impact of MDA depends on the efficacy of the antimalarial, coverage among the target population and local malaria epidemiology.^25,26^ High coverage (defined here as >80%) in the targeted population is essential to interrupt transmission.^26,27^ Mobilising entire communities to participate in MDA can be difficult to achieve because target communities are typically remote, have limited infrastructure, have low levels of literacy and can be highly mobile.^15–24^ To promote MDA uptake, community engagement and health education activities took place alongside TME.^20,25,28,29^

Recent research in Vietnam,^17^ Laos^21^ and the Thai–Myanmar border^16^ has investigated factors that affect coverage during MDA. Participation in community engagement activities that explained the rationale for MDA, greater knowledge of malaria and local social and cultural traditions, such as group decision making at the community or household level, were associated with higher coverage. Building on these studies, this article explores new areas that may influence the feasibility and acceptability of MDA, including seasonal timing, financial incentives and the MDA delivery model.

## Materials and methods

### Study design and study sites

This study took place in four villages in Battambang Province, western Cambodia, situated close to forests along the Thai border. The villages lie in an area of low, unstable *P. falciparum* malaria transmission where working in forested areas is an established risk factor for infection. MDAs were conducted in two villages in 2015 (herein termed ‘early MDA’) and in two villages in 2016 (‘delayed MDA’) as part of a randomised controlled clinical trial to establish MDA effectiveness and safety. In 2015, MDA was delivered at temporary clinics set up in each village centre to which participants travelled for 3 consecutive days for treatment; for these visits they received compensation for lost time. In 2016, MDA was delivered at both temporary clinics (again with compensation for participation) and during house-to-house visits by local volunteers (during which no compensation was offered).^11^

Malaria transmission in Cambodia is seasonal and typically peaks after the main rains have begun, although the arrival of the rains and the peak of malaria vary from year to year.^27^ The arrival and duration of the main rainy season also determine the type and timing of the agricultural activities in villages, which in turn affects the availability of the local population to participate in MDA. In both 2015 and 2016, MDA took place in July–September, during the rainy season.

### Questionnaire data collection

A questionnaire-based survey was conducted in late 2015, approximately 3 months after the completion of three rounds of MDA in villages randomised to ‘early MDA’. The questionnaire was designed to assess respondents’ sociodemographic characteristics; their knowledge, attitudes, perceptions and experiences regarding malaria; and their views of MDA. Household representatives (n=163) were approached at their homes by a Cambodian member of the research team who had not personally been part of delivering MDA. After obtaining consent, one adult (>18 y of age) from each household was interviewed and if the household head was absent, the researcher sought consent from and interviewed any other adult household member. If consent was given, the questionnaire was administered face-to-face at the respondent’s home. Questionnaires were conducted in Khmer and each took approximately 30 min to complete.

The questionnaire was adapted from a well-validated version used previously in Gambia,^30^ the Thai–Myanmar border,^16^ Vietnam^17^ and Laos.^21^ The questionnaire was translated, pretested and checked for clarity, language and comprehensibility by local staff. The questionnaire contained five parts (parts I and II: consent, ethnicity, language of interview and sociodemographic and economic characteristics; part III: health care–seeking behaviour; part IV: knowledge, practices, perceptions and attitudes towards malaria; part V: understanding, perceptions and attitudes towards MDA).

### Management and analysis of questionnaire data

Completed questionnaires were entered into a database and the consistency and accuracy of the data were cross-checked against source documents. Treatment records from the clinical trial were used to create an outcome variable ‘participation in MDA’, which was re-categorised as 1, ‘non-participation’ (zero rounds of MDA); 2, ‘incomplete/partial participation’ (one or two rounds) and 3, ‘complete participation’ (three rounds). Correlations between responses and participation categories were tested for using the χ^2^ test or Fisher’s exact test as appropriate. No adjustment was made to account for multiple comparisons. The 95% level was used to define statistically significant associations (p≤0.05) for further consideration. Analyses were conducted using SPSS Statistics for Windows, version 24.0 (IBM, Armonk, NY, USA).

Variables associated with participation by univariate analysis, such as malaria-related health care–seeking behaviour, knowledge and experience of MDA and attitudes towards MDA, were included in a logistic regression model to adjust for confounding. All significant predictors were explored and odds ratios (ORs) with confidence intervals (CIs) were calculated to predict complete (three round) participation.

### Qualitative data collection

The individual interviews and focus group discussions (FGDs) were conducted in late 2016 and early 2017 following MDA in two villages randomised to ‘delayed MDA’. From MDA treatment records, participation status was defined as complete, incomplete or refused, then, stratified according to these categories, individual respondents were selected at random. If the selected person was unavailable, the next person on the list replaced that participant. Respondents included villagers who participated in MDA (n=15), villagers who had refused to participate or did not complete the three rounds of antimalarial administration (n=5), health staff involved in community engagement (n=5) and village leaders (5 FGDs). Interviews and FGDs took place at or near the participants’ houses and were conducted in the Khmer language using a question guide, with observations recorded by a note-taker. All interviews and focus group discussions were audio recorded, verbatim transcribed and translated to English by a professional agency (Translation Services Cambodia, Phnom Penh). Sample sizes were subject to data collection reaching theoretical saturation, whereby no novel findings emerged from subsequent interviews.^31^

### Village calendar data collection

In early 2017, group meetings with village leaders and local stakeholders were held in each study village to prepare a calendar of yearly working, agricultural and migration patterns. Forest-acquired malaria is common in all these communities, so the timing of forest work was included in the calendar. Annual incidence records for *P. falciparum* and *Plasmodium vivax* were obtained from the Battambang Provincial Health Department. Climate data were obtained from the nearest meteorological station (Battambang).

## Results

### Questionnaire survey

#### Participation in MDA

Treatment records recorded during MDA were reviewed and among 163 respondents, 19 (11.7%) did not participate, 63 (38.7%) participated in one or two rounds and 81 (49.7%) completed all three rounds of MDA. A total of 13/19 (68.4%) non-participants did not take MDA because they were excluded on medical grounds (8 because of either acute illness or an underlying chronic condition, 4 pregnant women, 1 lactating mother) and 6/19 (31.6%) refused.

#### Sociodemographic characteristics of the respondents

The majority of respondents were Khmer (162/163 [99.4%]), were literate (129/163 [79.1%] could at least read Khmer), most were farmers (135/163 [82.8%]), the mean monthly household income was US$145 and approximately half of the respondents had a toilet at home (87/163 [53.4%]). Most respondents were non-migrants (132/163 [81.0%]) and almost all resided close to forested areas (<5 km; 161/163 [98.7%]). (Supplementary Table 1)

#### Health care–seeking behaviour

Approximately a quarter of respondents (40/163 [24.5%]) recalled having a fever episode within the previous 3 months, for which the majority sought treatment (38/40 [95.0%]). Among those who sought treatment, 19/38 (50.0%) attended a private clinic and 15/40 (37.5%) attended the nearest public health centre (Supplementary Table 2). Respondents who attended a health centre when they had a fever were more likely to complete all three MDA rounds compared with those who attended a local drug shop or private clinic (p=0.001).

#### Knowledge of malaria and MDA

Almost all respondents had heard about malaria (161/163 [98.8%]) and were aware of its symptoms: fever (157/163 [97.5%]), headache (136/163 [84.5%]) and chills (154/163 [95.7%]) (Supplementary Table 3). Almost all agreed with the statement ‘malaria is a big problem in my community’ (158/163 [96.9%]). Most respondents (154/163 [94.5%]) reported sleeping under a mosquito net the previous night. Most respondents (152/163 [93.3%]) knew the correct duration (3 days) for taking the first-line antimalarial treatment. In a question where multiple answers were possible, half the respondents (81/163 [49.7%]) expressed a preference to receive health information through village meetings (other popular answers were from a health care worker. 51/163 [31.3%]; by radio or television, 62/163 [38%] or from family members, 10/163 [6.1%]), and the people who mentioned village meetings were more likely to have completed three MDA rounds than those who did not (49/81 [60.5%]; p=0.02).

Most respondents agreed that a ‘villager can have malaria parasites in their body without being sick’ (130/163 [79.8%]), and those who agreed with the statement were more likely to complete all three MDA rounds (70/130 [53.8%]) compared with those who did not think this was possible (11/33 [33.3%]; p=0.043) (Supplementary Table 4). Respondents who volunteered during the implementation of the MDA, such as by attending meetings, assisting during mobilisation activities, bringing their family for treatment or visiting neighbouring houses (140/163 [85%]), were more likely to complete all three MDA rounds (76/140 [54.2%]) compared with those who did not (5/23 [21.7%]; p=0.004). A total of 148/163 (90.8%) respondents stated they would like to take antimalarials as part of MDA in the future.

#### Logistic regression model to explore factors affecting MDA uptake

Three factors were independently associated with the completion of all MDA rounds: seeking treatment at a government health centre for fever (adjusted odds ratio [aOR] 2.64 [CI 1.01 to 6.88]; p=0.04), knowledge of asymptomatic malaria (aOR 3.31 [CI 1.14 to 9.62]; p=0.02) and awareness of MDA (aOR 3.32 [CI 1.4 to 7.4]; p=0.004) (Table 1).

**Table 1.** Logistic regression of variables associated^a^ with completion of participation in MDA

Questionnaire responses	No MDA (n=19), n (%)	Incomplete (n=63), n (%)	Complete (n=81), n (%)	Odds ratio (95% CI)	p-Value	aOR^b^ (95% CI)	p-Value
Farmer as an occupation	12 (8.9)	53 (39.3)	70 (51.9)	1.7 (0.7 to 3.8)	0.22	1.3 (0.5 to 3.7)	0.61
I will go to health centre first (if I am sick)	7 (7.8)	27 (30)	56 (62.2)	3.2 (1.7 to 6.0)	<0.001	2.6 (1.0 to 6.9)	0.046
I prefer government health centres	9 (8.8)	31 (30.4)	62 (60.8)	3.4 (1.8 to 6.7)	<0.001	1.6 (0.6 to 4.4)	0.35
Road to health centre is good	9 (10.0)	42 (46.7)	39 (43.3)	1.9 (1.0 to 3.6)	0.04	1.5 (0.7 to 3.3)	0.35
I want to receive information from village meetings	8 (9.9)	24 (29.6)	49 (60.5)	2.4 (1.3 to 4.5)	0.007	1.7 (0.8 to 3.6)	0.2
Prevention of mosquito bite using hammock	10 (10.9)	28 (30.4)	54 (58.7)	2.3 (1.2 to 4.4)	0.009	2.1 (0.9 to 4.6)	0.07
I slept under a mosquito net last night	15 (9.7)	62 (40.3)	77 (50)	1.3 (0.32 to 4.83)	0.74	1.6 (0.3 to 8.3)	0.6
MDA medicines should be taken for 3 days	15 (9.9)	58 (38.2)	79 (52)	4.9 (1.0 to 23.3)	0.04	3.4 (0.5 to 21.3)	0.2
A healthy-looking person can have malaria parasite	16 (12.3)	44 (33.8)	70 (53.8)	2.3 (1.0 to 5.2)	0.03	0.7 (0.2 to 1.9)	0.46
Carriers do not become sick because they have few parasites	1 (3.2)	7 (22.6)	23 (74.2)	3.7 (1.5 to 8.8)	0.004	3.3 (1.1 to 9.6)	0.02
Apparently healthy people with parasites are dangerous	15 (12.1)	40 (32.3)	69 (55.6)	2.8 (1.3 to 6.1)	0.008	2.6 (0.9 to 7.4)	0.07
The entire community should be involved to eliminate malaria	14 (9.7)	56 (38.9)	74 (51.4)	1.8 (0.7 to 4.9)	0.23	0.9 (0.2 to 3.4)	0.86
I participated as a volunteer in the TME study	14 (10.0)	50 (35.7)	76 (54.3)	4.3 (1.5 to 12.2)	0.006	3.6 (1.0 to 13.2)	0.056
I have heard of MDA before	5 (6.3)	26 (32.5)	49 (61.3)	2.5 (1.3 to 4.7)	0.004	3.3 (1.5 to 7.5)	0.004
I would participate in MDA in the future	15 (9.5)	63 (39.9)	80 (50.6)	4.1 (0.5 to 37.5)	0.21	0.8 (0.03 to 14.9)	0.85
I would take the medicine offered in the MDA in the future	12 (8.1)	61 (41.2)	75 (50.7)	1.5 (0.5 to 4.5)	0.43	1.1 (0.3 to 4.3)	0.93

^a^By χ^2^ test for univariate analysis (see Supplementary Tables 1–4).

^b^OR adjusted for age and sex.

### Individual interviews and FGDs

#### Incentives and their absence

Generally, community members stated that the lack of a financial incentive did not influence their participation. Respondents identified eliminating malaria as the most important reason for participating; ultimately their health was their paramount consideration.

*R: They gave me [the incentive] every time. Oh, actually, they didn’t give it most recently…*

*[…]*

*I: And how did you feel about that?*

*R: It was fine!*

*[…]*

*I: Why did you still join?*

*R: I didn’t want to suffer from malaria! They are eliminating malaria*.

—Individual interview in village of Phnom Rei

#### Concerns about malaria

Recognising malaria as an important health hazard underpinned respondents’ reasons to participate in MDA. Participants’ concerns were linked to past experiences of malaria: they described bouts of malaria and febrile illness that they or their immediate family had suffered, mentioning experiences from years ago. Such concerns prompted the (reported) use of bed nets and wearing of long-sleeved/legged clothes to prevent mosquito bites, particularly in forests. Indeed, people who travelled to forests were considered particularly at risk of malaria and two respondents also referred specifically to people returning from the forest and causing the spread of malaria in villages.

#### Prevention through MDA

Respondents specifically mentioned prevention through taking drugs. Participants also reported a desire for the results of malaria tests to ensure that the disease had been ‘eliminated’ from their bodies. This suggests that MDA was sometimes considered as curative of existing infections and sometimes antimalarial prophylaxis.

*I: How can we protect ourselves from getting malaria?*

*R: We can take medicine.*

*[…]*

*I: What if we don’t have medicine to take?*

*R: If we don’t, we will still have malaria.*

—Focus group discussion in the hamlet of Ting Moung

#### Participation despite perceptions of side effects

The majority of respondents linked some sort of bodily complaint with the MDA. They described a plethora of perceived adverse events, including nausea, dizziness, sweating, malaise, trembling, fever, chest pains, lack of appetite, indigestion and heartburn, many of which—based on their description—had an unproven link to the MDA. In spite of this, most continued to participate and seemed content when study staff explained that their problems might not be related to the MDA.

*I: And how did you feel after taking that medicine?*

*R: After taking the medicine, I did not feel well; I had a headache and felt nauseous. It was like I had contracted malaria again! [Laugh]*

*[…]*

*I: But, you kept taking the medicine even though you did not feel well?*

*R: I continued to take it for the full three days.*

—Individual interview in the village of Phnom Rei

#### House-to-house delivery of MDA without incentives

The house-to-house visits were generally well received. Respondents liked that they could ask the study staff questions and this was seen as a gesture of care—as an expression of concern for their health—particularly by older respondents.

It was good because they came to our house, and we did not need to go to see other doctors, or if we wanted to know about anything, we could ask them. We did not need to ask the others. It helped us to save our gasoline rather than us travelling to see them.

—Individual interview in the commune of Ta Taok

In terms of centralised events, respondents were generally happy with the community meetings and the mobilisation events, although several described not being able to attend these because of flooding. Respondents described how various commitments, particularly travelling for work, limited involvement in the meetings and other activities. MDA was given as directly observed treatment except for a few occasions when a participant travelled away from the village during the MDA and was given the remaining medicine to complete or for a small number of participants who did not want to take medicine before work or travel. There was an indirect report of non-adherence, participants would tell the project staff that they wanted to take the tablets after meal times but then they would not do so.

#### Involving ethnic minorities

In 2015 the villages that received MDA were >99% ethnic Khmer, but in 2016 one of the villages had a large ethnic minority population. The respondents indicated that community members from the ethnic minorities did not present particular difficulties to achieving high levels of coverage. Study staff explained this in part by the approach of involving ethnic leaders in the community engagement and then with their agreement, other members of their community would also participate. The challenges were more related to the isolated nature of these settlements; access by road was sometimes limited and the lack of mobile phone coverage made it more difficult to inform villagers of changes to the study routine.

### Seasonal timing of MDA

**Figure 1.**
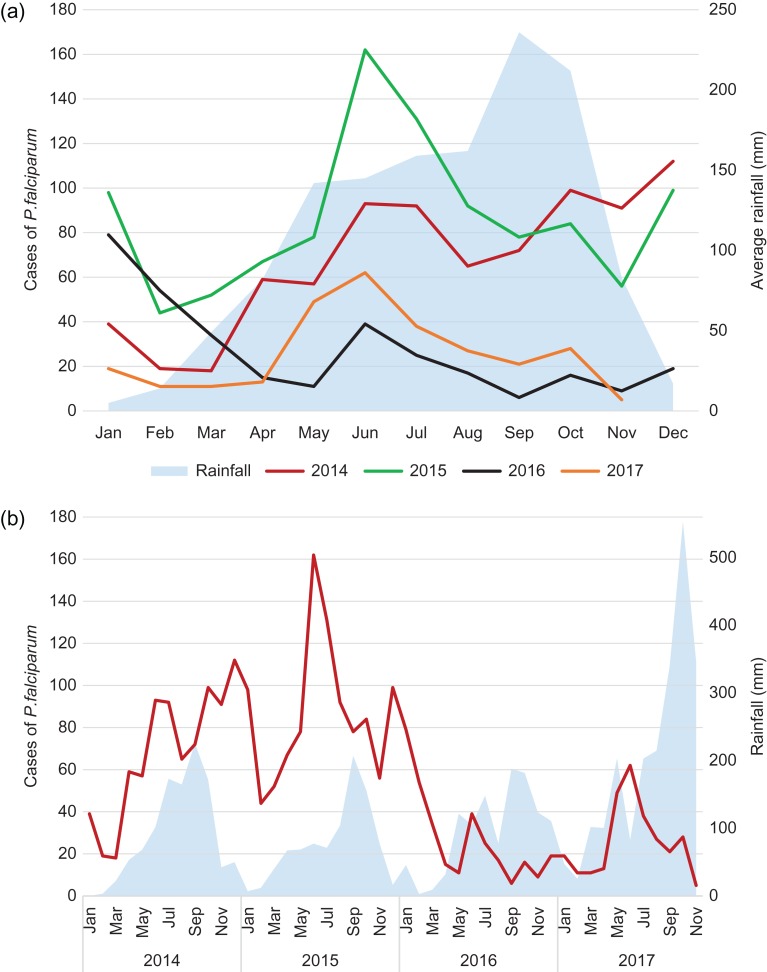
(a) *Plasmodium falciparum* and mixed *P. falciparum* and *Plasmodium vivax* cases in Battambang Province, 2014–2017, and average monthly rainfall. (b) *P. falciparum* and mixed *P. falciparum* and *P. vivax* cases in Battambang, 2014–2017, and yearly rainfall.

Epidemiological data were combined with village calendars (Supplementary Figures 2a–c) to highlight the advantages and disadvantages of conducting MDA during different seasons. In most years the main rainfall peaked between May and October. *P. falciparum* incidence was higher in the second half of the year but there was transmission throughout the year and an apparent decline in the total number of cases over the 4-y period that was likely unrelated to MDA (Figures 1a and 1b).

In both 2015 and 2016, MDA was given during July–September and the impact over the following 12 months met one World Health Organization definition of interruption of transmission, that is, <1/1000 cases of clinical *P. falciparum* per year. Despite this success, the effort needed to achieve high coverage during the rainy season was substantial.^24^ Village calendars and practical experience suggest that MDA is logistically easier to implement during the dry season, as residents are less busy with agricultural work. Most forest work occurs in the early wet season and it may be more difficult to get this higher-risk population to participate in MDA at that time (Table 2).

**Table 2.** Considerations for selecting the most appropriate time of year to conduct MDA

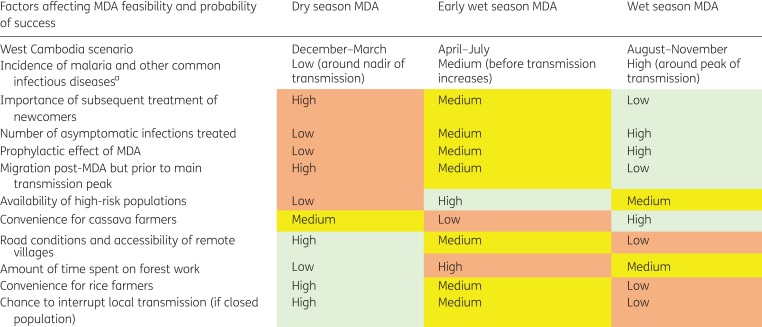

^a^Populations are more concerned about malaria during the peak of transmission and this may encourage participation. However, diseases such as common colds and diarrhoea are more prevalent during this time. This might encourage participation as people seek treatment, but conversely might lead to increased numbers of people being excluded from participation with MDA or the attribution of illness to an adverse reaction to medicines given during MDA.

Cells have been coloured according to discussions with the field team who implemented MDA: orange-red represents the least favourable timing, yellow intermediate and pale green most favourable.

## Discussion

This study explored factors that influence the acceptability and feasibility of MDA for malaria elimination that have not been explicitly addressed in previous research: incentives, delivery model and seasonal timing.^25^

Reasons provided by the respondents for participating in MDA were complex. Awareness of the existence of asymptomatic *P. falciparum* infections was associated with higher rates of participation, which has been observed elsewhere in the GMS where understanding the rationale for MDA correlated with participation.^15,17,21^ However, this may be a result of the fact that people who participated in the MDA were also more likely to have attended meetings where staff explained the study and the concept of asymptomatic malaria. Concerns about suffering from malaria and a desire to eliminate malaria were reported as motivating factors to participate in the MDA, and coverage did not drop when incentives were removed. The aim of eliminating malaria was described as more important than financial incentives, which reflects the findings from Laos.^22^

Despite logistical challenges in more isolated villages, coverage among ethnic minority groups was similar to that among the Khmer population, possibly explained by the close involvement of ethnic community leaders in meetings and during mobilisation. The meaningful involvement of community leaders to promote local ownership is a key element of effective community engagement.^20,25^ MDA uptake is subject to a range of influences and intertwined with the local social context. For example, in Laos, conformity within villages contributed to high levels of participation, whereas in Myanmar, community divisions led to low levels of coverage in some villages.^15,18,19,21^

House-to-house MDA was as acceptable as delivery at a central location and both approaches resulted in similarly high levels of coverage. Using a delivery point in the centre of the village meant that people working outside of the village arrived when it was convenient for them and also helped the MDA team stay organised. House-to-house MDA was facilitated by the involvement of popular, local volunteers familiar with their neighbours. House-to-house MDA was viewed favourably, mainly because of convenience and because it provided opportunities for community members to ask questions on a one-to-one basis. In either delivery model, directly observed treatment for MDA was important to achieve appropriate dosing and medical exclusions and accurate recordkeeping and to ensure that perceived adverse events could be managed (with re-treatment or supportive care as needed).^23^

In western Cambodia, communities where MDA might be targeted are most accessible in the dry season (locally November–March). Mathematical modelling suggests MDA during the late dry season has the best chance of interrupting transmission since parasite prevalence is lowest.^27^ During the dry season, residents may also find it easier to participate because there is less agricultural or forest work. For these reasons, MDA in these areas might best be conducted from February to early April, with activities completed by Khmer New Year. Dry season MDA requires the successful identification and treatment of newcomers who arrive in villages later in the year and may have malaria parasites that could reintroduce transmission.^32–34^ Therefore dry season MDA might necessitate a longer-term commitment by community volunteers. In contrast, MDA during the rainy season is logistically more challenging but treats more prevalent infections, any prophylactic effect of MDA (in this instance produced by the long half-life of piperaquine) would be greatest and effective population coverage (not undermined by subsequent movement) would be highest during the period when it is most important. However, even if MDA is given in the rainy season, the potential for subsequent population movement to reintroduce transmission implies that if it is practicable, MDA delivered over a larger geographic area may be the most effective.

### Strengths and limitations

The questionnaire was previously used in other countries and allows comparison of our results with other MDAs, but it did not permit triangulation of key questions against responses from interviews such as opinions on MDA delivered at home or centrally. Data collected during interviews and surveys could be subject to social desirability bias, yet coverage data from the clinical study demonstrated no effect on uptake of MDA following the removal of incentives.^11^ The communities studied may be representative of malarial villages in Battambang, but there are likely to be differences between these villages and those in other Cambodian provinces in terms of malaria epidemiology, socio-economics and agricultural practices.

## Conclusions

MDA given at a central location has logistical benefits but requires participants to come for treatment, whereas house-to-house delivery is easier for participations but places more work on volunteers. Either model of delivering treatment is feasible, or with sufficient mobilisation it may be possible to combine both. Selecting the most appropriate season to conduct MDA will necessitate planning the MDA together with local stakeholders. In western Cambodia, conducting MDA at the end of the dry season probably presents the most advantages for scaling up the strategy, but if this timing is adopted, preventing the re-importation of malaria will be critical.

## Supplementary data

Supplementary data are available at Transactions online (http://trstmh.oxfordjournals.org/).

## Supplementary Material

Supplementary Data

Supplementary Data
