# Supplementary material for: The feasibility and acceptability of mass drug administration for malaria in Cambodia: a mixed-methods study
Source: Trans R Soc Trop Med Hyg. 2018 Jun 16;112(6):264–71. doi: 10.1093/trstmh/try053 (PMC6044409; doi:10.1093/trstmh/try053)

**Supplementary Figure 2a: Yearly calendar of major activities in three villages near to forests that received mass drug administration during July-September 2015**


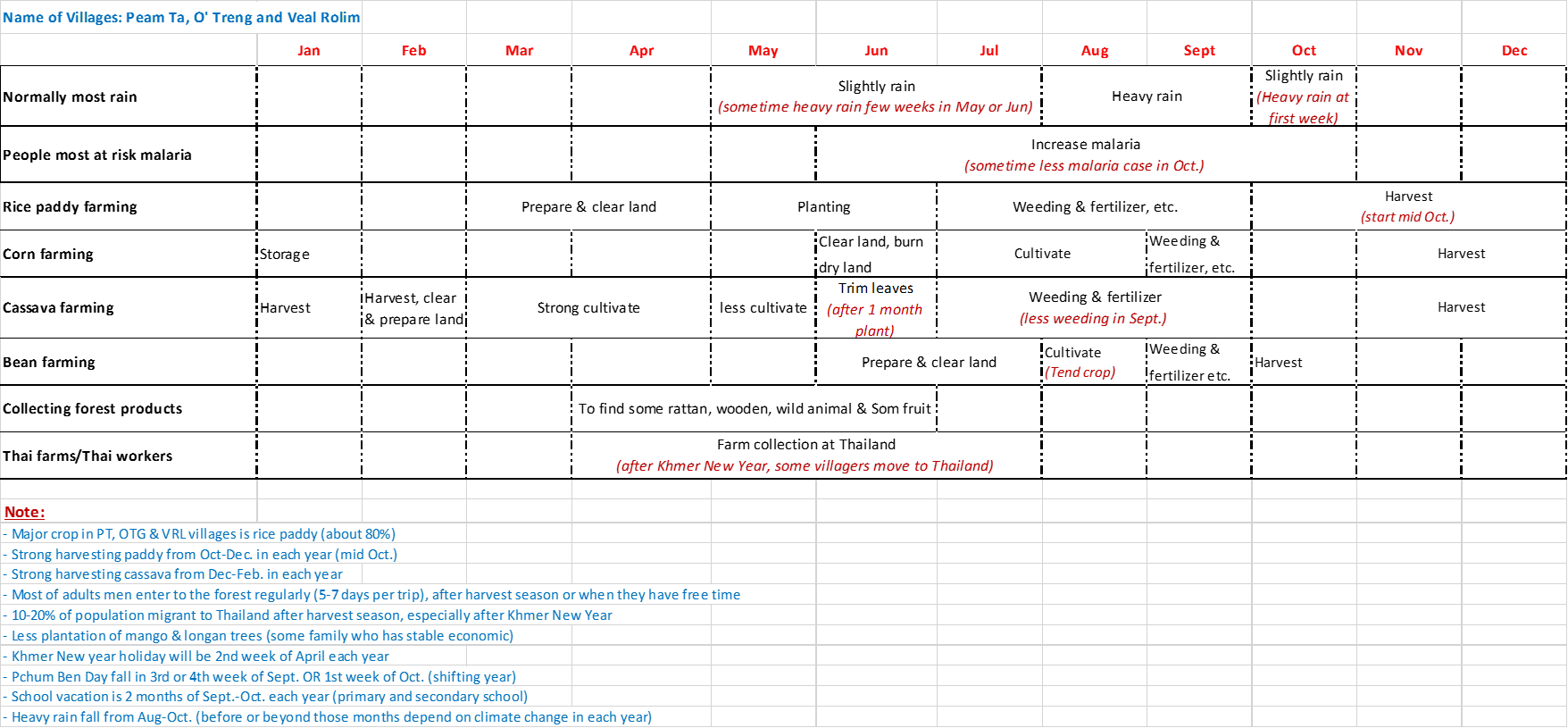


**Supplementary Figure 2b: Yearly calendar of major activities in one village situated aways from forests that received mass drug administration during July-September 2016**


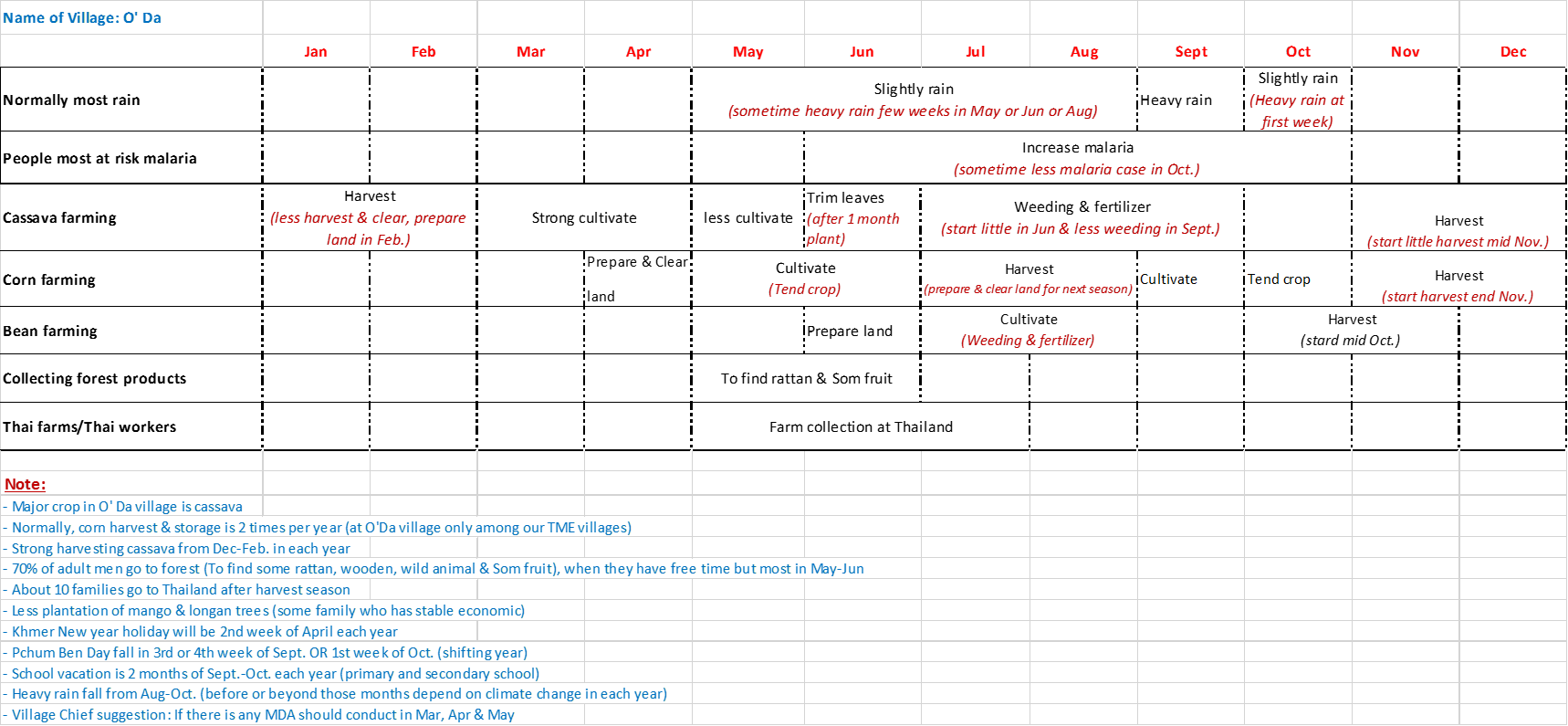


**Supplementary Figure 2c: Yearly calendar of major activities in three hamlets of one large village situated near to forests that received mass drug administration during July-September 2016**


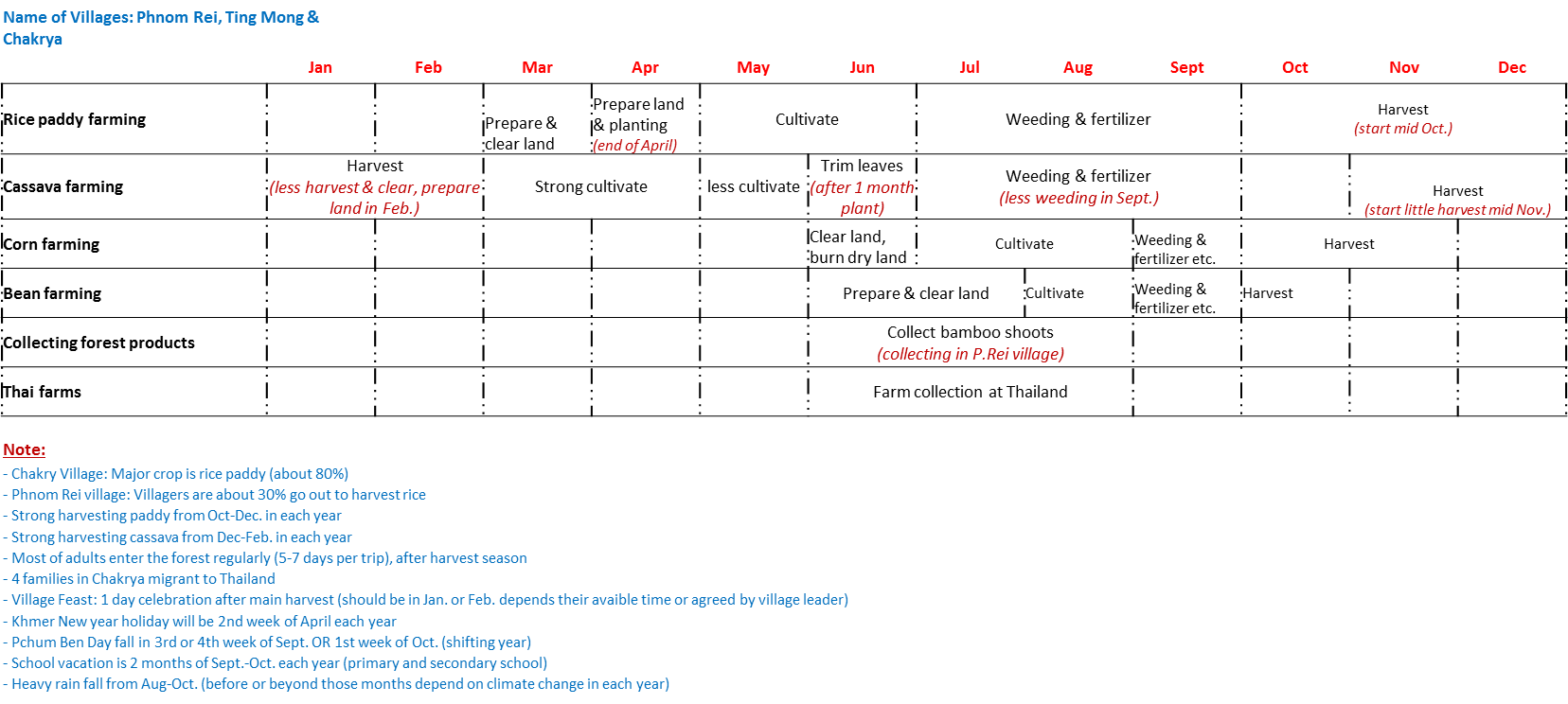

Supplement: Supplementary Data [file try053_supplementary_figures_2a,2b,2c.docx]
