# Supplementary material for: The feasibility and acceptability of mass drug administration for malaria in Cambodia: a mixed-methods study
Source: Trans R Soc Trop Med Hyg. 2018 Jun 16;112(6):264–71. doi: 10.1093/trstmh/try053 (PMC6044409; doi:10.1093/trstmh/try053)
Supplement: Supplementary Data [file try053_supplementary_tables_1,2,3,4.docx]

**Supplementary Tables 1-4**

**1. Quantitative analysis of post-MDA questionnaire responses in relation to participation status**

| **Table 1 Socio-demographic characteristics of the respondents in relation to participation in MDA (n=163)** | | | | | |
| --- | --- | --- | --- | --- | --- |
|  |  | **Participation** | |  |  |
| **Characteristics** | **None (n=19)** | **Incomplete (n=63)** | **Complete (n=81)** | **Total (n=163)** | **p-value** |
|  | **Number (%)** | **Number (%)** | **Number (%)** | **Number (%)** | |
| **Age group** |  |  |  |  |  |
| ≤ 34 | 5 (26.3) | 26 (41.3) | 30 (37) | 61 (37.4) | 0.59 |
| 35 to 49 years | 5 (26.3) | 10 (15.9) | 20 (24.7) | 35 (21.5) |  |
| ≥ 50 years | 9 (47.4) | 27 (42.9) | 31 (38.3) | 67 (41.1) |  |
| **Sex** |  |  |  |  |  |
| Female | 17 (89.5) | 43 (68.3) | 49 (60.5) | 109 (66.9) | 0.052 |
| Male | 2 (10.5) | 20 (31.7) | 32(39.5) | 54 (33.1) |  |
| **Ethnicity** |  |  |  |  |  |
| Khmer | 19 (100) | 62 (98.4) | 81 (100) | 162 (99.4) | 0.45 |
| Kachok | 0 | 1 (1.6) | 0 | 1 (0.6) |  |
| **Marital Status** |  |  |  |  |  |
| Not in relationship | 0 | 0 | 1 | 1 (0.6) |  |
| In relationship | 19 (100) | 63 (100) | 80 (98.8) | 162 (99.4) |  |
| **Literacy** |  |  |  |  |  |
| Can read and write | 15 (78.9) | 49 (77.8) | 61 (75.3) | 125 (76.7) | 0.56 |
| Can read but not write | 0 | 3 (4.8) | 1 (1.2) | 4 (2.5) |  |
| Cannot read and write | 4 (21.1) | 11 (17.5) | 19 (23.5) | 34 (20.9) |  |
| **Literacy Recoded** |  |  |  |  |  |
| Literate | 15 (78.9) | 52 (82.5) | 62 (76.5) | 129 (79.1) | 0.68 |
| Illiterate | 4 (21.1) | 11 (17.5) | 19 (23.5) | 34 (20.9) |  |
| **Level of education** |  |  |  |  |  |
| No education | 3 (15.8) | 11 (17.5) | 17 (21) | 31 (19) | 0.56 |
| Primary (≤6 years) | 11 (57.9) | 35 (55.6) | 43 (53.1) | 89 (54.6) |  |
| Above Primary (>6 years) | 5 (26.3) | 17 (27) | 21 (25.9) | 43 (26.4) |  |
| **Occupation** |  |  |  |  |  |
| Farmer | 12 (63.2) | 53 (84.1) | 70 (86.4) | 135 (82.8) | **0.05** |
| Non-farmers | 7 (36.8) | 10 (15.9) | 11 (13.6) | 28 (17.2) |  |
| **Monthly income** |  |  |  |  |  |
| ≤ 75 USD (Q1) | 4 (21.1) | 24 (38.1) | 26 (32.1) | 54 (33.1) | 0.06 |
| 75 to 120 USD (Q1 to Q2) | 3 (15.8) | 5 (7.9) | 20 (24.7) | 28 (17.2) |  |
| ≥ 120 USD (Q3) | 12 (63.2) | 34 (54) | 35 (43.2) | 81 (49.7) |  |
| Mean=146.52, Min=75, Max=992 | |  |  |  |  |
| Q1=74.28, Q2=120, Q3=185.7 | |  |  |  |  |
| **Daily expense (n=162)** |  |  |  |  |  |
| ≤ 2.5 USD (Q1) | 6 (31.6) | 19 (30.2) | 34 (42) | 59 (36.2) | 0.45 |
| 2.5 to 3.7 USD (Q2) | 4 (21.1) | 14 (22.2) | 20 (24.7) | 38 (23.3) |  |
| >3.7 USD (Q3) | 9 (47.4) | 30 (47.6) | 27 (33.3) | 66 (40.5) |  |
| Mean=4, Min=0.7, Max=25 | |  |  |  |  |
| Q1=2.5, Q2=3.7, Q3=4.95 | |  |  |  |  |
| **Income enough for living** |  |  |  |  |  |
| Yes | 11 (57.9) | 33 (52.4) | 45 (55.6) | 89 (54.6) | 0.77 |
| Don't know | 0 (0) | 1 (1.6) | 0 (0) | 1 (0.6) |  |
| NA | 1 (5.3) | 1 (1.6) | 1 (1.2) | 3 (1.8) |  |
| **What do you own?*** |  |  |  |  |  |
| House | 19 (100) | 62 (98.4) | 80 (98.8) | 161 (98.8) | 0.85 |
| Land | 17 (89.5) | 57 (90.5) | 72 (88.9) | 146 (89.6) | 0.95 |
| Motorbike | 15 (78.9) | 51 (81) | 61 (75.3) | 127 (77.9) | 0.71 |
| Bicycles | 7 (36.8) | 16 (25.4) | 14 (17.3) | 37 (22.7) | 0.15 |
| Tractor | 2 (10.5) | 5 (7.9) | 3 (3.7) | 10 (6.1) | 0.4 |
| Cars | 8 (42.1) | 17 (27) | 22 (27.2) | 47 (28.8) | 0.39 |
| Cattles | 11 (57.9) | 37 (58.7) | 36 (44.4) | 84 (51.5) | 0.19 |
| TV | 6 (31.6) | 15 (23.8) | 15 (18.5) | 36 (22.1) | 0.42 |
| Radio | 14 (73.7) | 53 (84.1) | 61 (75.3) | 128 (78.5) | 0.38 |
| Generators | 6 (31.6) | 17 (27) | 12 (14.8) | 35 (21.5) | 0.11 |
| **What are the walls of your house made of? *** | |  |  |  |  |
| Bamboo | 0 | 3 (4.8) | 9 (11.1) | 12 (7.4) | 0.14 |
| Wood | 17 (89.5) | 59 (93.7) | 73 (90.1) | 149 (91.4) | 0.71 |
| Concrete | 3 (15.8) | 0 | 0 | 3 (1.8) | **<0.001** |
| Brick | 2 (10.5) | 0 | 0 | 2 (1.2) | **<0.001** |
| Metal | 3 (15.8) | 10 (15.9) | 8 (9.9) | 21 (12.9) | 0.52 |
| **What is the roof of your house made of? *** | |  |  |  |  |
| Bamboo | 0 | 7 (11.1) | 11 (13.6) | 18 (11) | 0.23 |
| Wood | 6 (31.6) | 19 (30.2) | 25 (30.9) | 50 (30.7) | 0.99 |
| Mud | 0 | 1 (1.6) | 0 | 1 (0.6) | 0.45 |
| Concrete | 2 (10.5) | 0 | 1 (1.2) | 3 (1.8) | **0.01** |
| Plastic | 0 | 0 | 1 (1.2) | 1 (0.6) | 0.6 |
| Metal | 16 (84.2) | 51 (81) | 59 (72.8) | 126 (77.3) | 0.38 |
| Shingles | 4 (21.1) | 6 (9.5) | 10 (12.3) | 20 (12.3) | 0.4 |
| **What is the floor of your house made of?*** | |  |  |  |  |
| Bamboo | 1 (5.3) | 2 (3.2) | 5 (6.2) | 8 (4.9) | 0.7 |
| Wood | 16 (84.2) | 53 (84.1) | 67 (82.7) | 136 (83.4) | 0.97 |
| Mud | 1 (5.3) | 3 (4.8) | 5 (6.2) | 9 (5.5) | 0.93 |
| Concrete | 1 (5.3) | 6 (9.5) | 2 (2.5) | 9 (5.5) | 0.18 |
| Brick | 0 | 0 | 1 (1.2) | 1 (0.6) | 0.6 |
| Plastic | 0 | 1 (1.6) | 0 | 1 (0.6) | 0.45 |
| Tiles | 2 (10.5) | 0 | 4 (4.9) | 6 (3.7) | 0.07 |
| **Do you have a toilet at home?** | |  |  |  |  |
| Yes | 16 (84.2) | 39 (61.9) | 32 (39.5) | 87 (53.4) | **<0.001** |
| No | 3 (15.8) | 24 (38.1) | 49 (60.5) | 76 (46.6) |  |
| **Type of toilet (n=87)** |  |  |  |  |  |
| Flush Toilet | 12 (75) | 28 (71.8) | 23 (71.9) | 63 (72.4) | 0.86 |
| Pit Latrine | 4 (25) | 10 (25.6) | 9 (28.1) | 23 (26.4) |  |
| NA | 0 | 1 (2.6) | 0 | 1 (1.1) |  |
| **Migrated from another village** | |  |  |  |  |
| Yes | 1 (5.3) | 14 (22.2) | 16 (19.8) | 31 (19) | 0.24 |
| No | 18 (94.7) | 49 (77.8) | 65 (80.2) | 132 (81) |  |
| **Living in the village in years (n=31)** |  |  |  |  |  |
| ≤ 10 years | 0 | 10 (71.4) | 10 (62.5) | 20 (64.5) | 0.34 |
| 11 to 37 years | 1 (100) | 4 (28.6) | 4 (25) | 9 (29) |  |
| NA | 0 | 0 | 2 (12.5) | 2 (6.5) |  |
| **Distance to forest** |  |  |  |  |  |
| ≤ 1km | 9 (47.4) | 30 (47.6) | 41 (50.6) | 80 (49.1) | 0.47 |
| 1.5 to 5km | 9 (47.4) | 33 (52.4) | 39 (48.1) | 81 (49.7) |  |
| NA | 1 (5.3) | 0 | 1 (1.2) | 2 (1.2) |  |
| **Distance to rice field** |  |  |  |  |  |
| ≤ 0.8km | 5 (26.3) | 12 (19) | 31 (38.3) | 48 (29.4) | 0.15 |
| ≥ 1km | 12 (63.2) | 45 (71.4) | 45 (55.6) | 102 (62.6) |  |
| NA | 2 (10.5) | 6 (9.5) | 5 (6.2) | 13 (8) |  |
| **Frequency of visit to forest** | |  |  |  |  |
| ≤ every alternate day | 2 (10.5) | 17 (27) | 16 (19.8) | 35 (21.5) | 0.34 |
| ≥ every week to month | 3 (15.8) | 14 (22.2) | 17 (21) | 34 (20.9) |  |
| Not at all | 13 (68.4) | 27 (42.9) | 36 (44.4) | 76 (46.6) |  |
| NA | 1 (5.3) | 5 (7.9) | 12 (14.8) | 18 (11) |  |
| *Multiple answers were possible, calculations were made against "yes" and "no" and includes "yes" answers. | | | | | |
| p values were calculated using Chi square and Fisher exact test | | | |  |  |

| **Table 2 Health seeking behaviour of the respondents in relation to participation in MDA (n=163)** | | | | | |
| --- | --- | --- | --- | --- | --- |
|  |  | **Participation** |  |  |  |
| **Characteristics** | **None (n=19)** | **Incomplete (n=63)** | **Complete (n=81)** | **Total (n=163)** | **p-value** |
|  | **Number (%)** | **Number (%)** | **Number (%)** | **Number (%)** |  |
| **Fever in the past** |  |  |  |  |  |
| Yes | 5 (26.3) | 15 (23.8) | 20 (24.7) | 40 (24.5) | 0.97 |
| No | 14 (73.7) | 48 (76.2) | 61 (75.3) | 123 (75.5) |  |
| **If yes, did you seek treatment? (n=40)** | |  |  |  |  |
| Yes | 5 (100) | 15 (100) | 18 (90) | 38 (95) | 0.71 |
| No | 0 | 0 | 1 (5) | 1 (2.5) |  |
| NA | 0 | 0 | 1 (5) | 1 (2.5) |  |
| **If Yes, where did you seek for treatment? (n=38)** | | |  |  |  |
| Self-medicated | 0 | 1 (6.7) | 0 | 1 (2.6) | 0.1 |
| Local drug shop | 1 (20) | 1 (6.7) | 0 | 2 (5.3) |  |
| Private clinic | 2 (40) | 11 (73.3) | 6 (33.3) | 19 (50) |  |
| Nearest health centre | 2 (40) | 2 (13.3) | 11 (61.1) | 15 (39.5) |  |
| Other | 0 | 0 | 1 (5.6) | 1 (2.6) |  |
| **Where do you go first for treatment if you are sick?** | | |  |  |  |
| Self-medication | 1 (5.3) | 0 | 1 (1.2) | 2 (1.2) | **0.001** |
| Local drug shop | 1 (5.3) | 1 (1.6) | 5 (6.2) | 7 (4.3) |  |
| Private clinic | 8 (42.1) | 30 (47.6) | 11 (13.6) | 49 (30.1) |  |
| Nearest health centre | 7 (36.8) | 27 (42.9) | 56 (69.1) | 90 (55.2) |  |
| Other | 2 (10.5) | 5 (7.9) | 8 (9.9) | 15 (9.2) |  |
| **Which do you prefer?** | |  |  |  |  |
| Khmer Traditional medicine | 1 (5.3) | 3 (4.8) | 3 (3.7) | 7 (4.3) | **0.008** |
| Government Health centres | 9 (47.4) | 31 (49.2) | 62 (76.5) | 102 (62.6) |  |
| Private clinic | 9 (47.4) | 29 (46) | 16 (19.8) | 54 (33.1) |  |
| **Distance to health centre from home** | |  |  |  |  |
| ≤5km | 10 (52.6) | 33 (52.4) | 35 (43.2) | 78 (47.9) | 0.82 |
| >6km | 7 (36.8) | 25 (39.7) | 38 (46.9) | 70 (42.9) |  |
| NA | 2 (10.5) | 5 (7.9) | 8 (9.9) | 15 (9.2) |  |
| **Mode of transport to health centre** | |  |  |  |  |
| Motorbike | 16 (84.2) | 52 (82.5) | 72 (88.9) | 140 (85.9) | 0.8 |
| Other (walk, bicycle, cart, tractor, car, other) | 2 (10.5) | 9 (14.3) | 7 (8.6) | 18 (11) |  |
| NA | 1 (5.3) | 2 (3.2) | 2 (2.5) | 5 (3.1) |  |
| **Road to health centre is in good condition** | | |  |  |  |
| Yes | 9 (47.4) | 42 (66.7) | 39 (48.1) | 90 (55.2) | **0.018** |
| No | 7 (36.8) | 20 (31.7) | 39 (48.1) | 66 (40.5) |  |
| NA | 3 (15.8) | 1 (1.6) | 3 (3.7) | 7 (4.3) |  |
| **Satisfied with the health centre** | |  |  |  |  |
| Yes | 13 (68.4) | 51 (81) | 68 (84) | 132 (81) | 0.51 |
| No | 3 (15.8) | 7 (11.1) | 6 (7.4) | 16 (9.8) |  |
| NA | 3 (15.8) | 5 (7.9) | 7 (8.6) | 15 (9.2) |  |
| **Can get anti-malarial over the counter (OTC)** | |  |  |  |  |
| No | 18 (94.7) | 62 (98.4) | 79 (97.5) | 159 (97.5) | 0.66 |
| Don't know | 1 (5.3) | 1 (1.6) | 2 (2.5) | 4 (2.5) |  |
| **Do you prefer OTC medication?** | |  |  |  |  |
| Yes | 0 | 0 | 1 (1.2) | 1 (0.6) | 0.81 |
| No | 19 (100) | 62 (98.4) | 78 (96.3) | 159 (97.5) |  |
| Don't know | 0 | 1 (1.6) | 2 (2.5) | 3 (1.8) |  |
| p values were calculated using Chi square and Fisher exact test | | | |  |  |

| **Table 3 knowledge on malaria of the respondents in relation to participation in MDA (n=163)** | | | | | |
| --- | --- | --- | --- | --- | --- |
|  |  | **Participation** |  |  |  |
| **Characteristics** | **None (n=19)** | **Incomplete (n=63)** | **Complete (n=81)** | **Total (n=163)** | **p-value** |
|  | **Number (%)** | **Number (%)** | **Number (%)** | **Number (%)** | |
| **Heard of malaria** |  |  |  |  |  |
| Yes | 19 (100) | 61 (96.8) | 81 (100) | 161 (98.8) | 0.52 |
| No | 0 | 1 (1.6) | 0 | 1 (0.6) |  |
| Don't know | 0 | 1 (1.6) | 0 | 1 (0.6) |  |
| **Symptoms of malaria (n=161)*** | |  |  |  |  |
| Fever | 19 (100) | 58 (95.1) | 80 (98.8) | 157 (97.5) | 0.28 |
| Headache | 16 (84.2) | 50 (82) | 70 (86.4) | 136 (84.5) | 0.76 |
| Muscle pain | 9 (47.4) | 26 (42.6) | 40 (49.4) | 75 (46.6) | 0.72 |
| Vomiting | 0 | 8 (13.1) | 8 (9.9) | 16 (9.9) | 0.24 |
| Chills | 18 (94.7) | 58 (95.1) | 78 (96.3) | 154 (95.7) | 0.92 |
| Sweating | 4 (21.1) | 6 (9.8) | 11 (13.6) | 21 (13) | 0.43 |
| Diarrhoea | 0 | 1 (1.6) | 0 | 1 (0.6) | 0.43 |
| Sore throat | 0 | 0 | 1 (1.2) | 1 (0.6) | 0.6 |
| Jaundice | 0 | 2 (3.3) | 3 (3.7) | 5 (3.1) | 0.7 |
| **If you have heard of malaria, where did you receive the information from? (n=161)*** | | | | | |
| Radio | 3 (15.8) | 9 (14.8) | 16 (19.8) | 28 (17.4) | 0.72 |
| Television | 4 (21.1) | 7 (11.5) | 19 (23.5) | 30 (18.6) | 0.18 |
| Newspaper | 0 | 1 (1.6) | 1 (1.2) | 2 (1.2) | 0.85 |
| Families | 4 (21.1) | 11 (18) | 18 (22.2) | 33 (20.5) | 0.82 |
| Village meeting | 6 (31.6) | 19 (31.1) | 33 (40.7) | 58 (36.0) | 0.45 |
| Health worker | 7 (36.8) | 19 (31.1) | 31 (38.3) | 57 (35.4) | 0.67 |
| Banners | 3 (15.8) | 6 (9.8) | 8 (9.9) | 17 (10.6) | 0.73 |
| Don't know | 7 (36.8) | 28 (45.9) | 23 (28.4) | 58 (36.0) | 0.13 |
| **how do you wish to receive information *** | |  |  |  |  |
| Radio | 3 (15.8) | 8 (12.7) | 20 (24.7) | 31 (19) | 0.17 |
| Television | 4 (21.1) | 8 (12.7) | 19 (23.5) | 31 (19) | 0.25 |
| Newspaper | 0 | 1 (1.6) | 0 | 1 (0.6) | 0.45 |
| Families | 2 (10.5) | 5 (7.9) | 3 (3.7) | 10 (6.1) | 0.4 |
| Village meeting | 8 (42.1) | 24 (38.1) | 49 (60.5) | 81 (49.7) | **0.02** |
| Health worker | 2 (10.5) | 22 (34.9) | 27 (33.3) | 51 (31.3) | 0.11 |
| Banners | 1 (5.3) | 2 (3.2) | 7 (8.6) | 10 (6.1) | 0.39 |
| Entertainment | 2 (10.5) | 3 (4.8) | 9 (11.1) | 14 (8.6) | 0.38 |
| **Malaria is transmitted via*** | |  |  |  |  |
| Water | 1 (5.3) | 6 (9.5) | 5 (6.2) | 12 (7.4) | 0.69 |
| Soil | 0 | 4 (6.3) | 4 (4.9) | 8 (4.9) | 0.53 |
| Forest | 0 | 4 (6.3) | 4 (4.9) | 8 (4.9) | 0.53 |
| Germ | 0 | 2 (3.2) | 2 (2.5) | 4 (2.5) | 0.73 |
| Mosquito | 17 (89.5) | 61 (96.8) | 76 (93.8) | 154 (94.5) | 0.44 |
| Uncleaned Surrounding | 1 (5.3) | 3 (4.8) | 7 (8.6) | 11 (6.7) | 0.63 |
| **Prevention of mosquito bite at home*** | | |  |  |  |
| Mosquito net | 18 (94.7) | 63 (100) | 78 (96.3) | 159 (97.5) | 0.25 |
| Burning wood | 0 | 6 (9.5) | 11 (13.6) | 17 (10.4) | 0.2 |
| Using Hammock | 1 (5.3) | 2 (3.2) | 7 (8.6) | 10 (6.1) | 0.39 |
| Repellents | 2 (10.5) | 7 (11.1) | 12 (14.8) | 21 (12.9) | 0.76 |
| Smoking | 0 | 0 | 1 (1.2) | 1 (0.6) | 0.6 |
| Oral medicine | 0 | 1 (1.6) | 1 (1.2) | 2 (1.2) | 0.85 |
| Sleeve | 9 (47.4) | 28 (44.4) | 38 (46.9) | 75 (46) | 0.95 |
| **Prevention of mosquito bite at farm*** | | |  |  |  |
| Mosquito net | 10 (52.6) | 33 (52.4) | 43 (53.1) | 86 (52.8) | 0.99 |
| Burning wood | 10 (52.6) | 37 (58.7) | 46 (56.8) | 93 (57.1) | 0.89 |
| Using Hammock | 10 (52.6) | 28 (44.4) | 54 (66.7) | 92 (56.4) | **0.02** |
| Repellents | 4 (21.1) | 19 (30.2) | 23 (28.4) | 46 (28.2) | 0.74 |
| Smoking | 0 | 3 (4.8) | 5 (6.2) | 8 (4.9) | 0.53 |
| Oral medicine | 0 | 2 (3.2) | 3 (3.7) | 5 (3.1) | 0.7 |
| Sleeve | 10 (52.6) | 32 (50.8) | 52 (64.2) | 94 (57.7) | 0.24 |
| Nothing | 0 | 1 (1.6) | 0 | 1 (0.6) | 0.45 |
| **Slept under the mosquito net last night** | | |  |  |  |
| Yes | 15 (78.9) | 62 (98.4) | 77 (95.1) | 154 (94.5) | **0.007** |
| No | 3 (15.8) | 1 (1.6) | 4 (4.9) | 8 (4.9) |  |
| Don't know | 1 (5.3) | 0 | 0 | 1 (0.6) |  |
| **How often you slept under the net (n=154)** | | |  |  |  |
| Everyday | 15 (100) | 61 (98.4) | 77 (100) | 153 (99.4) | 0.47 |
| Rarely | 0 | 1 (1.6) | 0 | 1 (0.6) |  |
| **Number of mosquito nets you possess** | | |  |  |  |
| ≤ 3 | 14 (73.7) | 42 (66.7) | 57 (70.4) | 113 (69.3) | 0.81 |
| > 4 | 5 (26.3) | 21 (33.3) | 24 (29.6) | 50 (30.7) |  |
| **Have impregnated nets** | |  |  |  |  |
| Yes | 19 (100) | 60 (95.2) | 80 (98.8) | 159 (97.5) | 0.3 |
| No | 0 | 3 (4.8) | 1 (1.2) | 4 (2.5) |  |
| **Mosquito net available for family member** | | |  |  |  |
| Yes | 17 (89.5) | 52 (82.5) | 74 (91.4) | 143 (87.7) | 0.44 |
| No | 1 (5.3) | 9 (14.3) | 6 (7.4) | 16 (9.8) |  |
| NA | 1 (5.3) | 2 (3.2) | 1 (1.2) | 4 (2.5) |  |
| **Do you get malaria if any family member has malaria** | | | | |  |
| Yes | 16 (84.2) | 50 (79.4) | 70 (86.4) | 136 (83.4) | 0.56 |
| No | 2 (10.5) | 12 (19) | 10 (12.3) | 24 (14.7) |  |
| Don't know | 1 (5.3) | 1 (1.6) | 1 (1.2) | 3 (1.8) |  |
| **If Yes, how? (n=136)*** | |  |  |  |  |
| Touch | 0 | 0 | 1 (1.4) | 1 (0.7) | 0.62 |
| Air | 0 | 0 | 1 (1.4) | 1 (0.7) | 0.62 |
| Mosquito bite | 16 (100) | 48 (96) | 67 (95.7) | 131 (96.3) | 0.7 |
| Water | 0 | 0 | 2 (2.9) | 2 (1.5) | 0.38 |
| Food | 0 | 0 | 2 (2.9) | 2 (1.5) | 0.38 |
| **How would you know if somebody has malaria?*** | | |  |  |  |
| Blood test | 18 (94.7) | 60 (95.2) | 76 (93.8) | 154 (94.5) | 0.93 |
| Will develop fever | 1 (5.3) | 12 (19) | 18 (22.2) | 31 (19) | 0.23 |
| Will go to see health worker | 0 | 1 (1.6) | 1 (1.2) | 2 (1.2) | 0.85 |
| Has visited forest before | 0 | 0 | 2 (2.5) | 2 (1.2) | 0.35 |
| **Malaria is a deadly disease** | |  |  |  |  |
| Yes | 19 (100) | 61 (96.8) | 79 (97.5) | 159 (97.5) | 0.26 |
| No | 0 | 0 | 2 (2.5) | 2 (1.2) |  |
| NA | 0 | 2 (3.2) | 0 | 2 (1.2) |  |
| **Scared of malaria** |  |  |  |  |  |
| Yes | 19 (100) | 58 (92.1) | 80 (98.8) | 157 (96.3) | 0.24 |
| No | 0 | 4 (6.3) | 1 (1.2) | 5 (3.1) |  |
| NA | 0 | 1 (1.6) | 0 | 1 (0.6) |  |
| **If scared, because (n=157)*** | |  |  |  |  |
| It can take life | 12 (63.2) | 39 (67.2) | 58 (72.5) | 109 (69.4) | 0.65 |
| Causes poor health | 3 (15.8) | 3 (5.2) | 4 (5) | 10 (6.4) | 0.2 |
| Causes extra expenses | 3 (15.8) | 2 (3.4) | 5 (6.3) | 10 (6.4) | 0.16 |
| Cannot work | 0 | 4 (6.9) | 4 (5) | 8 (5.1) | 0.49 |
| **Healthy person can have malaria** | |  |  |  |  |
| Yes | 13 (68.4) | 42 (66.7) | 63 (77.8) | 118 (72.4) | **0.05** |
| No | 6 (31.6) | 12 (19) | 7 (8.6) | 25 (15.3) |  |
| Don't know | 0 | 9 (14.3) | 11 (13.6) | 20 (12.3) |  |
| **If Yes, is presence of malaria dangerous? (n=118)** | | | |  |  |
| Yes | 12 (92.3) | 38 (90.5) | 61 (96.8) | 111 (94.1) | 0.36 |
| No | 0 | 2 (4.8) | 2 (3.2) | 4 (3.4) |  |
| Don't know | 1 (7.7) | 1 (2.4) | 0 | 2 (1.7) |  |
| NA | 0 | 1 (2.4) | 0 | 1 (0.8) |  |
| **Can malaria be cured by medicine?** | |  |  |  |  |
| Yes | 19 (100) | 62 (98.4) | 81 (100) | 162 (99.4) | 0.45 |
| No | 0 | 1 (1.6) | 0 | 1 (0.6) |  |
| **If Yes, how many days should medicine be taken? (n=162)** | | | |  |  |
| 3 days | 15 (78.9) | 58 (92.1) | 79 (97.5) | 152 (93.3) | **0.015** |
| > 3 days | 2 (10.5) | 4 (6.3) | 2 (2.5) | 8 (4.9) |  |
| NA | 2 (10.5) | 1 (1.6) | 0 | 2 (1.8) |  |
| **Would you complete the dose, if you feel better earlier?** | | | |  |  |
| Yes | 17 (89.5) | 60 (95.2) | 77 (95.1) | 154 (94.5) | 0.18 |
| No | 1 (5.3) | 3 (4.8) | 3 (3.7) | 7 (4.3) |  |
| Don't know | 1 (5.3) | 0 | 0 | 1 (0.6) |  |
| NA | 0 | 0 | 1 (1.2) | 1 (0.6) |  |
| **Have you ever had malaria before?** | | |  |  |  |
| Yes | 12 (63.2) | 44 (69.8) | 65 (80.2) | 121 (74.2) | 0.31 |
| No | 7 (36.8) | 18 (28.6) | 16 (19.8) | 41 (25.2) |  |
| Don't know | 0 | 1 (1.6) | 0 | 1 (0.6) |  |
| **If Yes, when did you have last episode? (n=121)** | | |  |  |  |
| Few months ago | 0 | 2 (4.5) | 3 (4.6) | 5 (4.1) | 0.87 |
| More than a year ago | 12 (100) | 39 (88.6) | 58 (89.2) | 109 (90.1) |  |
| I don't remember/Don't know | 0 | 3 (6.8) | 3 (4.6) | 6 (5) |  |
| NA | 0 | 0 | 1 (1.5) | 1 (0.8) |  |
| **If Yes, how did you know that you get malaria last time? (n=121)*** | | | | |  |
| Through blood test | 9 (75) | 38 (86.4) | 56 (86.2) | 103 (85.1) | 0.58 |
| Health worker informed me after blood test | 1 (91.7) | 4 (9.1) | 7 (10.8) | 12 (9.9) | 0.94 |
| Health worker informed me without blood test | 0 | 1 (2.3) | 1 (1.5) | 2 (1.7) | 0.85 |
| My illness looked like malaria | 0 | 2 (4.5) | 6 (9.2) | 8 (6.6) | 0.39 |
| Witchcraft/traditional healer diagnosed it | 1 (8.3) | 0 | 0 | 1 (0.8) | 0.01 |
| **Where did you go for treatment when you had malaria? (n=121)** | | | | |  |
| Traditional healer | 1 (8.3) | 2 (4.5) | 2 (3.1) | 5 (4.1) | 0.64 |
| Health centre/hospital | 6 (50) | 18 (40.9) | 31 (47.7) | 55 (45.5) |  |
| Self-medicated | 0 | 0 | 3 (4.6) | 3 (2.5) |  |
| Other# | 5 (41.7) | 24 (54.5) | 29 (44.6) | 58 (47.9) |  |
| **If Yes, how many days did you take medicine? (n=121)** | | | |  |  |
| 3 days | 7 (58.3) | 30 (68.2) | 57 (87.7) | 94 (77.7) | 0.08 |
| > 3 days | 2 (16.7) | 9 (20.5) | 6 (9.2) | 17 (14) |  |
| Did not take it | 0 | 1 (2.3) | 0 | 1 (0.8) |  |
| Don't remember | 2 (16.7) | 3 (6.8) | 2 (3.1) | 7 (5.8) |  |
| NA | 1 (8.3) | 1 (2.3) | 0 | 2 (1.7) |  |
| **Health centres are capable of treating malaria** | | |  |  |  |
| Yes | 13 (68.4) | 45 (71.4) | 66 (81.5) | 124 (76.1) | 0.31 |
| No | 2 (10.5) | 2 (3.2) | 3 (3.7) | 7 (4.3) |  |
| Don't know | 4 (21.1) | 11 (17.5) | 10 (12.3) | 25 (15.3) |  |
| NA | 0 | 5 (7.9) | 2 (2.5) | 7 (4.3) |  |
| *Multiple answers were possible, calculations were made against "yes" and "no" and includes "yes" answers. | | | | | |
| p values were calculated using Chi square and Fisher exact test | | | |  |  |
| #Included 38 VMWs out of total 58 | | | | | |

| **Table 4 knowledge and attitudes towards MDA of the respondents in relation to participation in MDA (n=163)** | | | | | |
| --- | --- | --- | --- | --- | --- |
|  |  | **Participation** | |  |  |
| **Characteristics** | **None (n=19)** | **Incomplete (n=63)** | **Complete (n=81)** | **Total (n=163)** | **p-value** |
|  | **Number (%)** | **Number (%)** | **Number (%)** | **Number (%)** | |
| **Malaria is a big problem in my community** | | |  |  |  |
| Yes | 18 (94.7) | 62 (98.4) | 78 (96.3) | 158 (96.9) | 0.42 |
| No | 0 | 1 (1.6) | 2 (2.5) | 3 (1.8) |  |
| Don’t know | 1 (5.3) | 0 | 1 (1.2) | 2 (1.2) |  |
| **A villager can have malaria parasites in his/her body without being sick** | | | |  |  |
| Yes | 16 (84.2) | 44 (69.8) | 70 (86.4) | 130 (79.8) | **0.043** |
| No | 3 (15.8) | 19 (30.2) | 11 (13.6) | 33 (20.2) |  |
| **If Yes, why are these people not sick? (n=130)*** | | |  |  |  |
| They are healthy/strong | 11 (68.8) | 33 (75) | 48 (68.6) | 92 (70.8) | 0.75 |
| They have immunity | 3 (18.8) | 4 (9.1) | 6 (8.6) | 13 (10) | 0.45 |
| They have very little parasite | 1 (6.3) | 7 (15.9) | 23 (32.9) | 31 (23.8) | **0.025** |
| Other | 3 (18.8) | 8 (18.2) | 7 (10) | 18 (13.8) | 0.39 |
| **If Yes, are these people dangerous?** | |  |  |  |  |
| Yes | 15 (93.8) | 40 (90.9) | 69 (98.6) | 124 (95.4) | **0.04** |
| No | 0 | 2 (4.5) | 0 | 2 (1.5) |  |
| Don't know | 0 | 2 (4.5) | 1 (1.4) | 3 (2.3) |  |
| NA | 1 (6.3) | 0 | 0 | 1 (0.8) |  |
| **If Yes, why are they dangerous? (n=124)*** | | |  |  |  |
| They can transmit to others | 6 (40) | 16 (40) | 31 (44.9) | 53 (42.7) | 0.85 |
| They can become sick in future | 6 (40) | 15 (37.5) | 27 (39.1) | 48 (38.7) | 0.98 |
| Don't know | 0 | 0 | 3 (4.3) | 3 (2.4) | 0.29 |
| **A healthy person with parasitaemia can transmit the infection to others** | | | |  |  |
| Yes | 16 (84.2) | 55 (87.3) | 75 (92.6) | 146 (89.6) | 0.38 |
| No | 1 (5.3) | 6 (9.5) | 4 (4.9) | 11 (6.7) |  |
| Don't know | 2 (10.5) | 2 (3.2) | 2 (2.5) | 6 (3.7) |  |
| **If Yes, what should be done to these people? (n=146)*** | | | |  |  |
| Provide them malaria medicine | 7 (43.8) | 21 (38.2) | 37 (49.3) | 65 (44.5) | 0.44 |
| Nothing | 0 | 1 (1.8) | 2 (2.7) | 3 (2.1) | 0.78 |
| Send to health centre | 6 (37.5) | 16 (29.1) | 30 (40) | 52 (35.6) | 0.44 |
| Don’t know | 0 | 4 (7.3) | 1 (1.3) | 5 (3.4) | 0.13 |
| **If Yes, should we provide medicine to all the villagers? (n=146)** | | | | |  |
| Yes | 16 (100) | 53 (96.4) | 71 (94.7) | 140 (95.9) | 0.9 |
| No | 0 | 1 (1.8) | 2 (2.7) | 3 (2.1) |  |
| Don't know | 0 | 1 (1.8) | 2 (2.7) | 3 (2.1) |  |
| **If Yes, why? (n=140)*** |  |  |  |  |  |
| To cure all people | 1 (6.3) | 12 (22.6) | 14 (19.7) | 27 (19.3) | 0.34 |
| To eliminate malaria from the village | 6 (37.5) | 13 (24.5) | 29 (40.8) | 48 (34.3) | 0.16 |
| To prevent malaria transmission in the village | 6 (37.5) | 17 (32.1) | 23 (32.4) | 46 (32.9) | 0.91 |
| To prevent us from malaria | 1 (6.3) | 13 (24.5) | 11 (15.5) | 25 (17.9) | 0.18 |
| **Have you heard of current malaria elimination in your village?** | | | |  |  |
| Yes | 16 (84.2) | 53 (84.1) | 74 (91.4) | 143 (87.7) | 0.53 |
| No | 3 (15.8) | 9 (14.3) | 7 (8.6) | 19 (11.7) |  |
| NA | 0 | 1 (1.6) | 0 | 1 (0.6) |  |
| **If Yes, what did you hear about it? (n=143)*** | | |  |  |  |
| Blood test | 3 (18.8) | 5 (9.4) | 11 (14.9) | 19 (13.3) | 0.53 |
| MDA | 1 (6.3) | 8 (15.1) | 14 (18.9) | 23 (16.1) | 0.44 |
| Health education | 9 (56.3) | 23 (43.4) | 30 (40.5) | 62 (43.4) | 0.51 |
| **Do you think malaria can be eliminated from your community** | | | |  |  |
| Yes | 15 (78.9) | 56 (88.9) | 74 (91.4) | 145 (89) | 0.41 |
| No | 2 (10.5) | 5 (7.9) | 5 (6.2) | 12 (7.4) |  |
| Don't know | 2 (10.5) | 2 (3.2) | 1 (1.2) | 5 (3.1) |  |
| NA | 0 | 0 | 1 (1.2) | 1 (0.6) |  |
| **If Yes, how? (n=145)*** |  |  |  |  |  |
| By giving medicine to all | 11 (73.3) | 43 (76.8) | 55 (74.3) | 109 (75.2) | 0.93 |
| By using mosquito nets | 6 (40) | 19 (33.9) | 37 (50) | 62 (42.8) | 0.18 |
| By taking regular medicine | 1 (6.7) | 2 (3.6) | 7 (9.5) | 10 (6.9) | 0.42 |
| By cleaning the surrounding | 0 | 7 (12.5) | 10 (13.5) | 17 (11.7) | 0.32 |
| **Entire community involvement is essential to eliminate malaria (n=145)** | | | | |  |
| Yes | 14 (93.3) | 56 (100) | 74 (100) | 144 (99.3) | **0.01** |
| NA | 1 (6.7) | 0 | 0 | 1 (0.7) |  |
| **Did you participate as a volunteer in TME?** | | |  |  |  |
| Yes | 14 (73.7) | 50 (79.4) | 76 (93.8) | 140 (85.9) | **0.004** |
| No | 4 (21.1) | 13 (20.6) | 5 (6.2) | 22 (13.5) |  |
| Don't know | 1 (5.3) | 0 | 0 | 1 (0.6) |  |
| **If Yes, why? (n=140)*** |  |  |  |  |  |
| I want to make my community free from malaria | 7 (50) | 29 (58) | 40 (52.6) | 76 (54.3) | 0.79 |
| I want to help my community | 3 (21.4) | 8 (16) | 8 (10.5) | 19 (13.6) | 0.45 |
| Malaria is a big problem in my community | 3 (21.4) | 6 (12) | 24 (31.6) | 33 (23.6) | **0.04** |
| **If No, why? (n=22)** |  |  |  |  |  |
| I am busy | 2 (50) | 5 (38.5) | 1 (20) | 8 (36.4) | 0.52 |
| I haven't understood about this program | 1 (25) | 1 (7.7) | 1 (20) | 3 (13.6) |  |
| Malaria is not a big problem | 0 | 1 (7.7) | 0 | 1 (4.5) |  |
| Other | 1 (25) | 0 | 0 | 1 (4.5) |  |
| Don't know | 0 | 3 (23.1) | 1 (20) | 4 (18.2) |  |
| NA | 0 | 3 (23.1) | 2 (40) | 5 (22.7) |  |
| **Would you participate in MDA as a volunteer?** | | |  |  |  |
| Yes | 19 (100) | 60 (95.2) | 78 (96.3) | 157 (96.3) | 0.67 |
| No | 0 | 3 (4.8) | 2 (2.5) | 5 (3.1) |  |
| NA | 0 | 0 | 1 (1.2) | 1 (0.6) |  |
| **Have you heard of MDA before the current campaign?** | |  |  |  |  |
| Yes | 5 (26.3) | 26 (41.3) | 49 (60.5) | 80 (49.1) | **0.002** |
| No | 13 (68.4) | 37 (58.7) | 32 (39.5) | 82 (50.3) |  |
| Don't know | 1 (5.3) | 0 | 0 | 1 (0.6) |  |
| **If Yes, where did you hear about it? (n=80)*** | | |  |  |  |
| Health personnel | 1 (20) | 6 (22.2) | 17 (34.7) | 24 (29.6) | 0.46 |
| Friends/family/villagers | 1 (20) | 0 | 7 (14.3) | 8 (10) | 0.1 |
| Radio/TV/Newspaper | 4 (80) | 25 (96.2) | 48 (98) | 77 (96.3) | 0.13 |
| **If Yes, have you taken part in MDA before? (n=80)** | | | |  |  |
| Yes | 1 (20) | 4 (15.4) | 16 (32.7) | 21 (26.3) | 0.53 |
| No | 4 (80) | 20 (76.9) | 30 (61.2) | 54 (67.5) |  |
| NA | 0 | 2 (7.7) | 3 (6.1) | 5 (6.3) |  |
| **Do you agree with the idea of MDA?** | |  |  |  |  |
| Yes | 18 (94.7) | 63 (100) | 81 (100) | 162 (99.4) | **0.022** |
| Don't know | 1 (5.3) | 0 | 0 | 1 (0.6) |  |
| **If Yes, why? (n=162)** |  |  |  |  |  |
| It protects us from malaria, | 1 (5.6) | 2 (3.2) | 1 (1.2) | 4 (2.5) | 0.47 |
| I want community to be free from malaria, | 4 (22.2) | 6 (9.5) | 10 (12.3) | 20 (12.3) |  |
| Other | 13 (72.2) | 55 (87.3) | 70 (86.4) | 138 (85.2) |  |
| **Would you participate again in MDA in future?** | | |  |  |  |
| Yes | 15 (78.9) | 63 (100) | 80 (98.8) | 158 (96.9) | **<0.001** |
| No | 3 (15.8) | 0 | 0 | 3 (1.8) |  |
| Don’t know | 1 (5.3) | 0 | 1 (1.2) | 2 (1.2) |  |
| **If Yes, why? (n=158)** |  |  |  |  |  |
| I want my community free from malaria | 3 (20) | 5 (7.9) | 11 (13.8) | 19 (12) | 0.53 |
| Other | 12 (80) | 58 (92.1) | 68 (85) | 138 (87.3) |  |
| NA | 0 | 0 | 1 (1.3) | 1 (0.6) |  |
| **Are you scared of blood test?** | |  |  |  |  |
| Yes | 6 (31.6) | 19 (30.2) | 21 (25.9) | 46 (28.2) | 0.06 |
| No | 11 (57.9) | 43 (68.3) | 60 (74.1) | 114 (69.9) |  |
| Don't know | 1 (5.3) | 0 | 0 | 1 (0.6) |  |
| NA | 1 (5.3) | 1 (1.6) | 0 | 2 (1.2) |  |
| **If Yes, why? (n=46)*** |  |  |  |  |  |
| We lose energy | 0 | 1 (5.3) | 1 (4.8) | 2 (4.3) | 0.85 |
| It is painful | 2 (33.3) | 3 (15.8) | 8 (38.1) | 13 (28.3) | 0.28 |
| It can bring other diseases | 1 (16.7) | 0 | 1 (4.8) | 2 (4.3) | 0.21 |
| We run out of blood | 0 | 1 (5.3) | 0 | 1 (2.2) | 0.48 |
| People perceive that they sell blood | 0 | 1 (5.3) | 0 | 1 (2.2) | 0.48 |
| Scared of needle | 2 (33.3) | 4 (21.1) | 3 (14.3) | 9 (19.6) | 0.57 |
| **Would you take medicine for MDA?** | |  |  |  |  |
| Yes | 12 (63.2) | 61 (96.8) | 75 (92.6) | 148 (90.8) | **<0.001** |
| No | 4 (21.1) | 2 (3.2) | 6 (7.4) | 12 (7.4) |  |
| NA | 3 (15.8) | 0 | 0 | 3 (1.8) |  |
| **If No, why? (n=12)** |  |  |  |  |  |
| I only take medicine when I am sick | 0 | 0 | 2 (33.3) | 2 (16.7) | 0.42 |
| I am scared of medicine side effects | 0 | 0 | 1 (16.7) | 1 (8.3) |  |
| I do not like to take medicine | 0 | 0 | 1 (16.7) | 1 (8.3) |  |
| Other (have a co-morbid condition) | 4 (100) | 2 (100) | 2 (33.3) | 8 (66.7) |  |
| *Multiple answers were possible, calculations were made against "yes" and "no" and includes "yes" answers. | | | | | |
| p values were calculated using Chi square and Fisher exact test | | | |  |  |
